# Supplementary material for: SiGra: single-cell spatial elucidation through an image-augmented graph transformer
Source: Nat Commun. 2023 Sep 12;14:5618. doi: 10.1038/s41467-023-41437-w (PMC10497630; doi:10.1038/s41467-023-41437-w)
Supplement: Supplementary file 1 — Supplementary File [file 41467_2023_41437_MOESM1_ESM.pdf]

SUPPLEMENTARY INFORMATION

SiGra: single-cell spatial elucidation through image-augmented graph transformer

Supplementary Figures ---------------- 3-11

Supplementary Notes ---------------- 12-19

Supplementary References ---------------- 20

Table of Contents

Supplementary Fig. 1: Illustration of the multi-head graph transformer layer in SiGra. *3*

Supplementary Fig. 2: SiGra enhances spatial gene expression data from different platforms. *4*

Supplementary Fig. 3: Visualization and evaluation of spatial domains identified by different methods in 6 of the total 12 DLPFC slices. *5*

Supplementary Fig. 4: Visualization and evaluation of spatial domains identified by different methods in the other 6 of the total 12 DLPFC slices. *6*

Supplementary Fig. 6: Examination of the effects of autofluorescence on SiGra's performance. Number of cropped images: 12 in each case.. *8*

Supplementary Fig. 7: Grid-based hyper-parameter fine turning... *9*

Supplementary Fig. 8: Comprehensive benchmarking and ablation studies. *10*

Supplementary Fig. 9: Comparisons with MUSE and STAGATE based on simulation data. . *11*

Supplementary Note 1: SiGra improves the quality of single-cell spatial transcriptomics data *12*

Supplementary Note 2: Interrogation of layer enriched gene markers in enhanced data *13*

Supplementary Note 3: Reveal spatial domains in single-cell spatial profiles *14*

Supplementary Note 4: Impact of autofluorescence signals on SiGra *15*

Supplementary Note 5: Hyperparameter tuning of SiGra model *15*

Supplementary Note 6: Comprehensive benchmarking, ablation studies, and simulation studies.. *16*

Supplementary References.. *20*

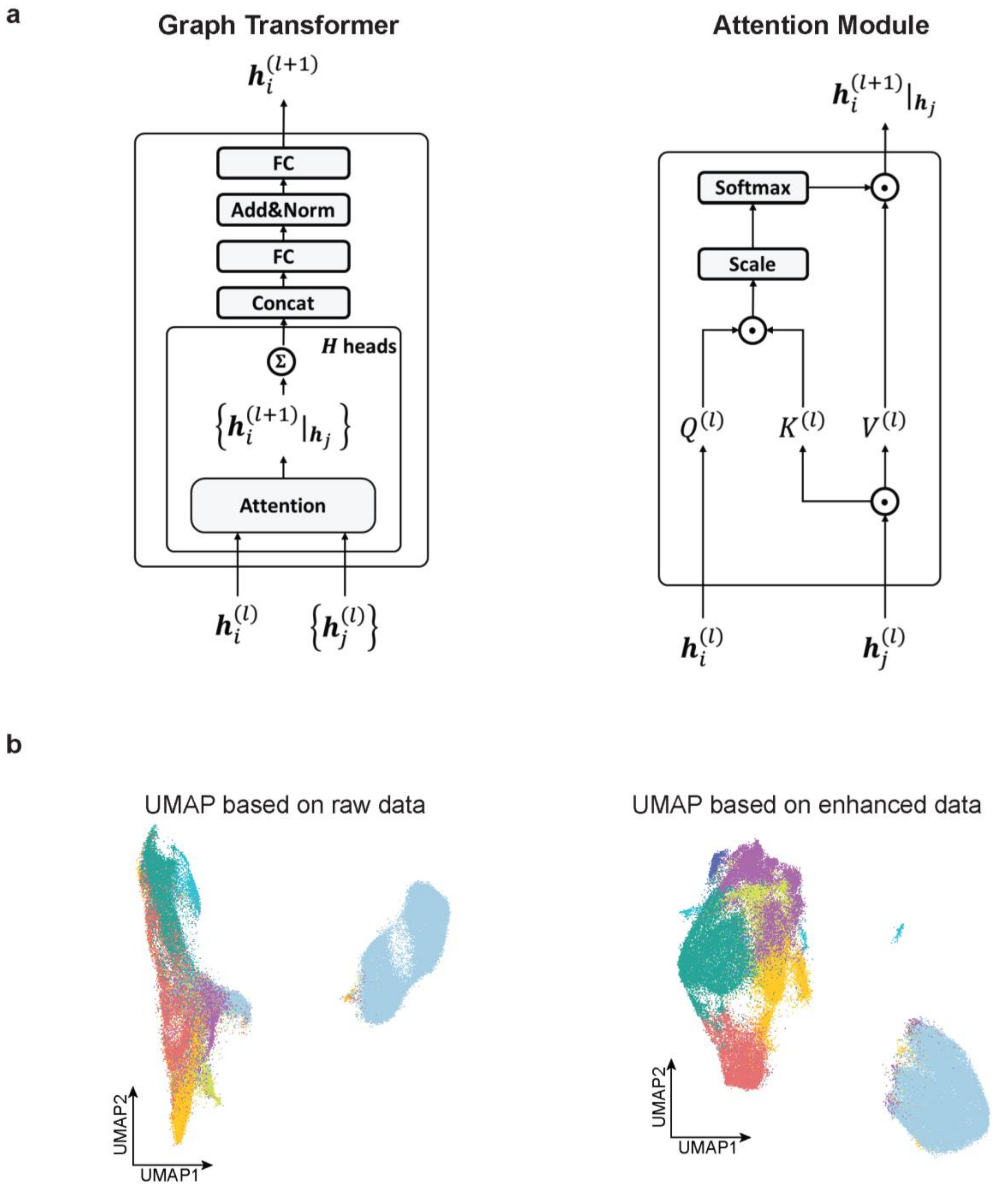

Supplementary Fig. 1: Illustration of the multi-head graph transformer layer in SiGra. a, the overall architecture of a graph transformer layer, and the attention module in the graph transformer. **b,** UMAP visualizations of raw data and the enhanced data of all cells.

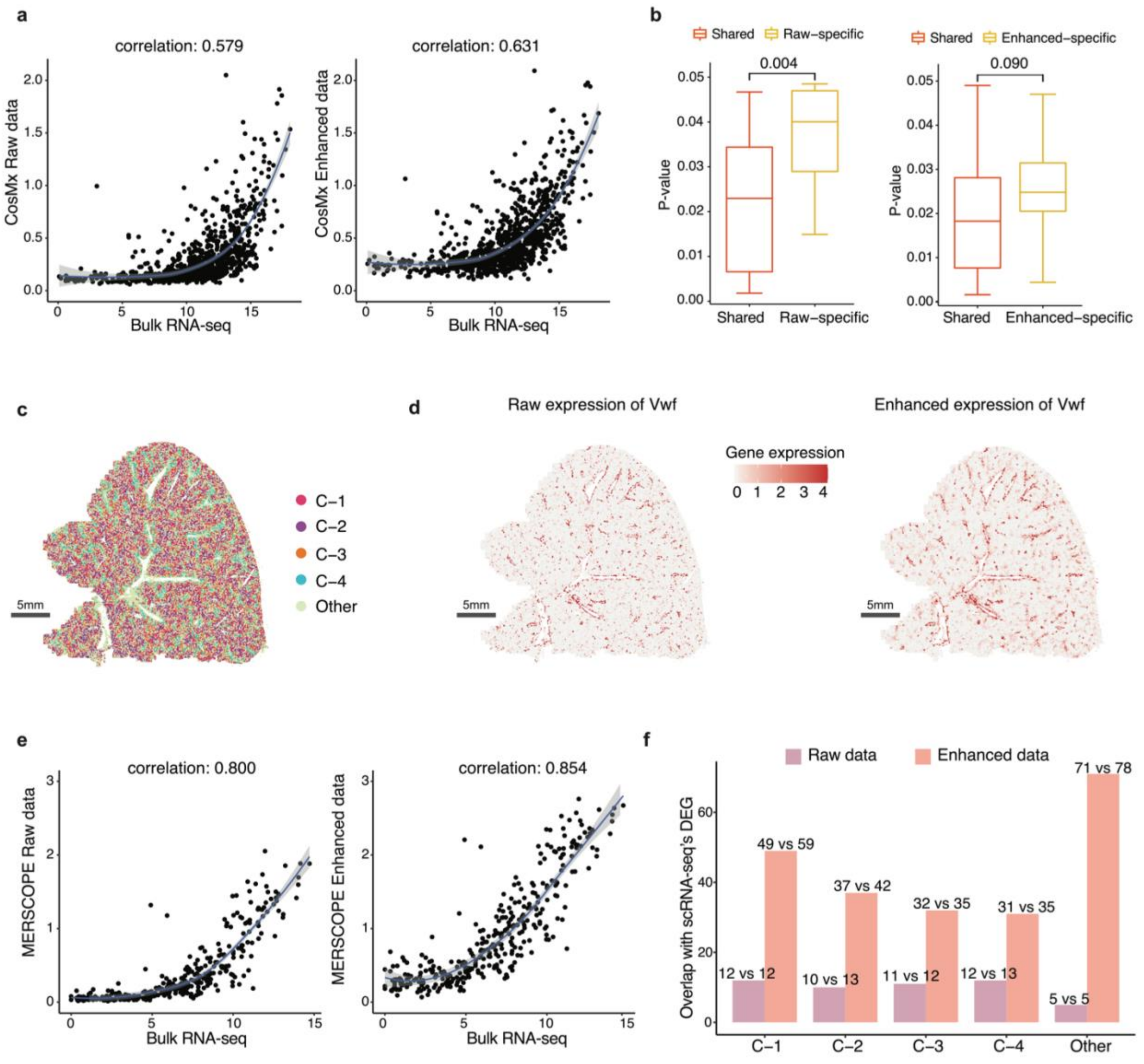

Supplementary Fig. 2: SiGra enhances spatial gene expression data from different platforms. a, Comparison of raw and enhanced data with bulk RNA-seq of lung cancer patient samples. **b,** Differences of enhanced-specific and raw-specific L-R pairs from shared L-R pairs (Number of L-R pairs in each group: shared: 28; raw-specific: 14; enhanced-specific: 27). In the boxplot, the center line, box limits and whiskers denote the median, upper and lower quartiles, and 1.5× interquartile range, respectively. **c,** Spatial visualization of all cell clusters in the single-cell spatial data from mouse liver tissue. **d,** Spatial visualization of the raw expressions and the enhanced expressions of *Vwf*. **e,** Comparison of enhanced data with bulk RNA-seq of mouse liver samples. **f,** Evaluation of DEGs using single-cell RNA-seq data of mouse liver samples.

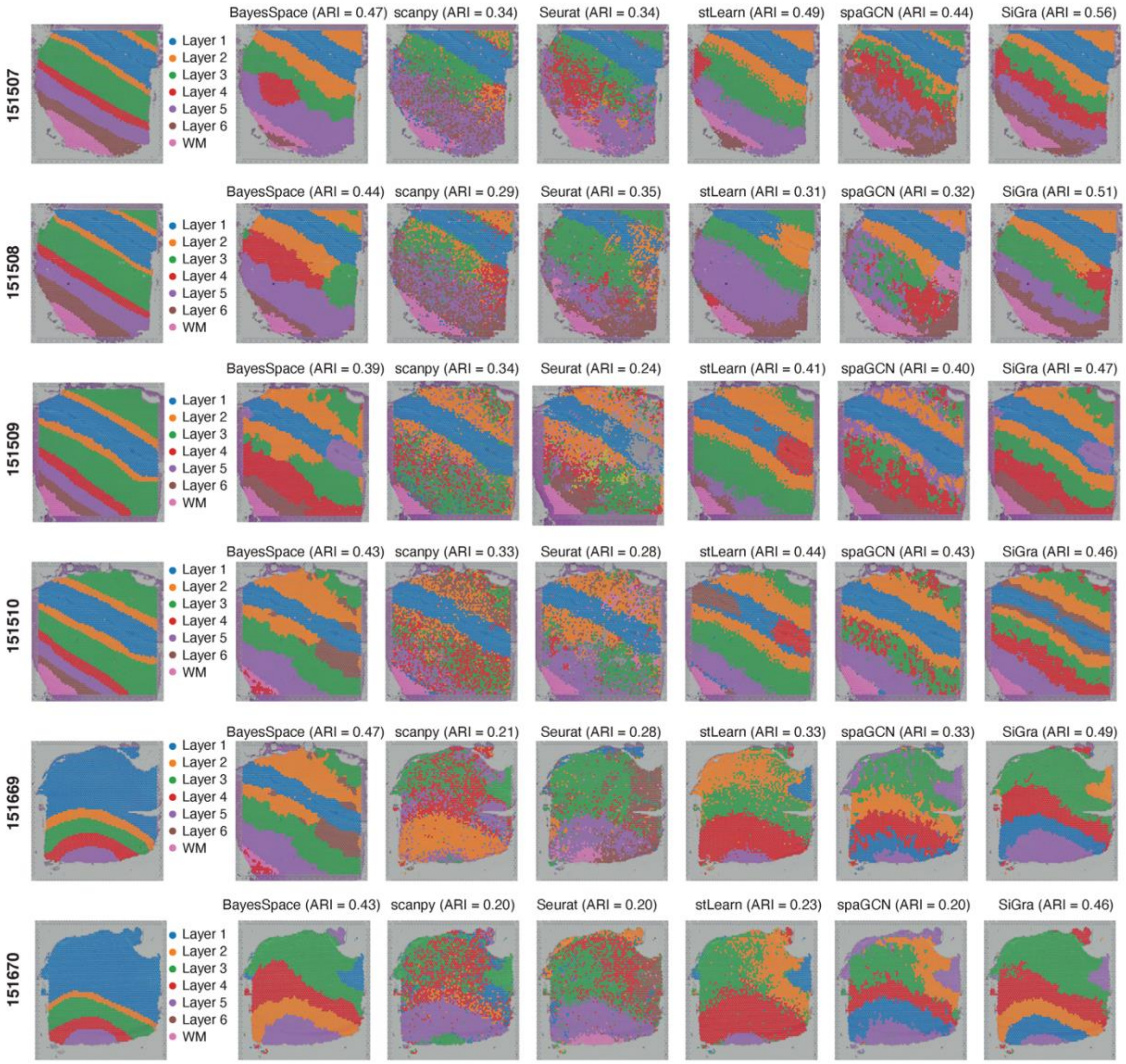

Supplementary Fig. 3: Visualization and evaluation of spatial domains identified by different methods in 6 of the total 12 DLPFC slices.

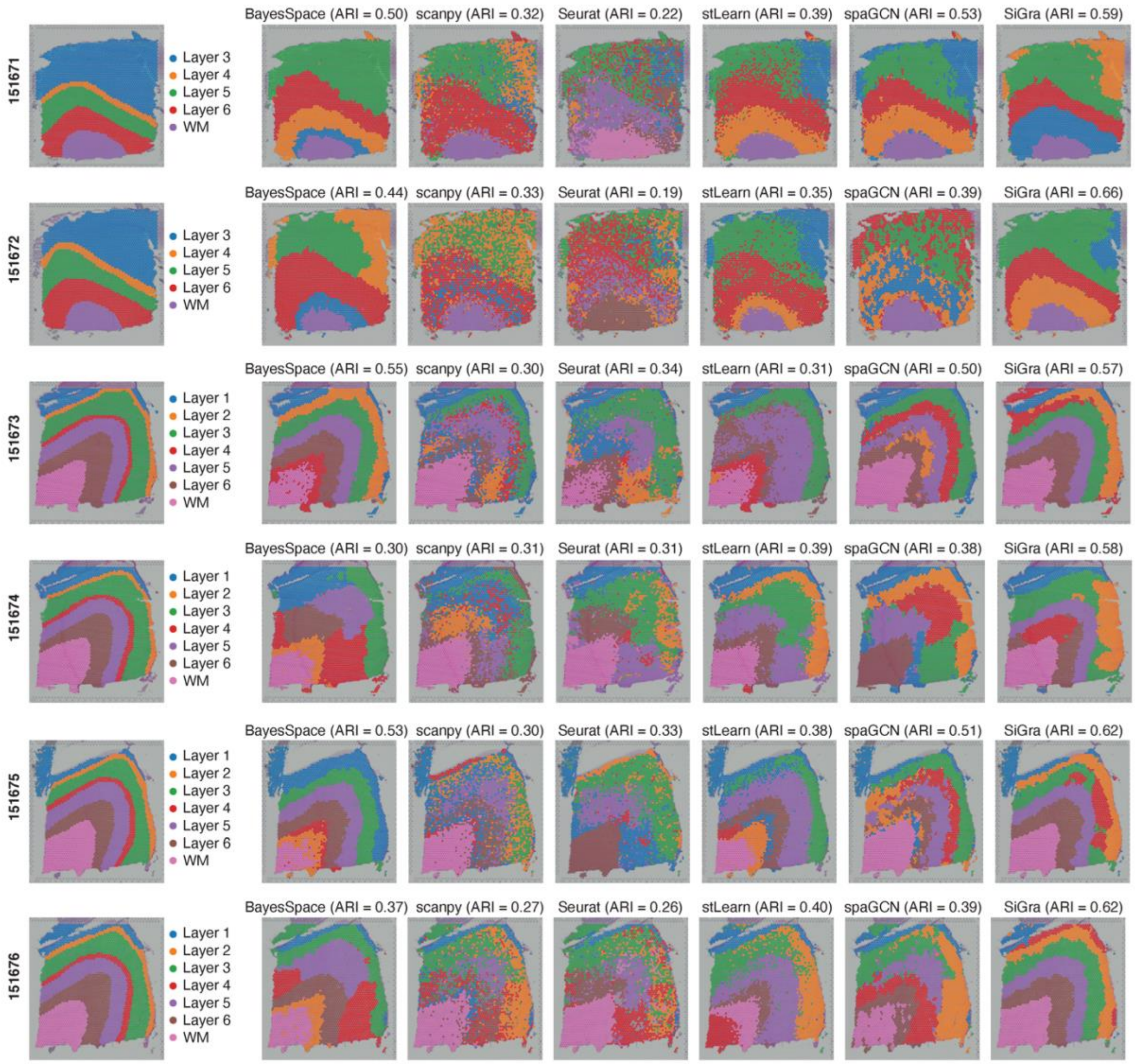

Supplementary Fig. 4: Visualization and evaluation of spatial domains identified by different methods in the other 6 of the total 12 DLPFC slices.

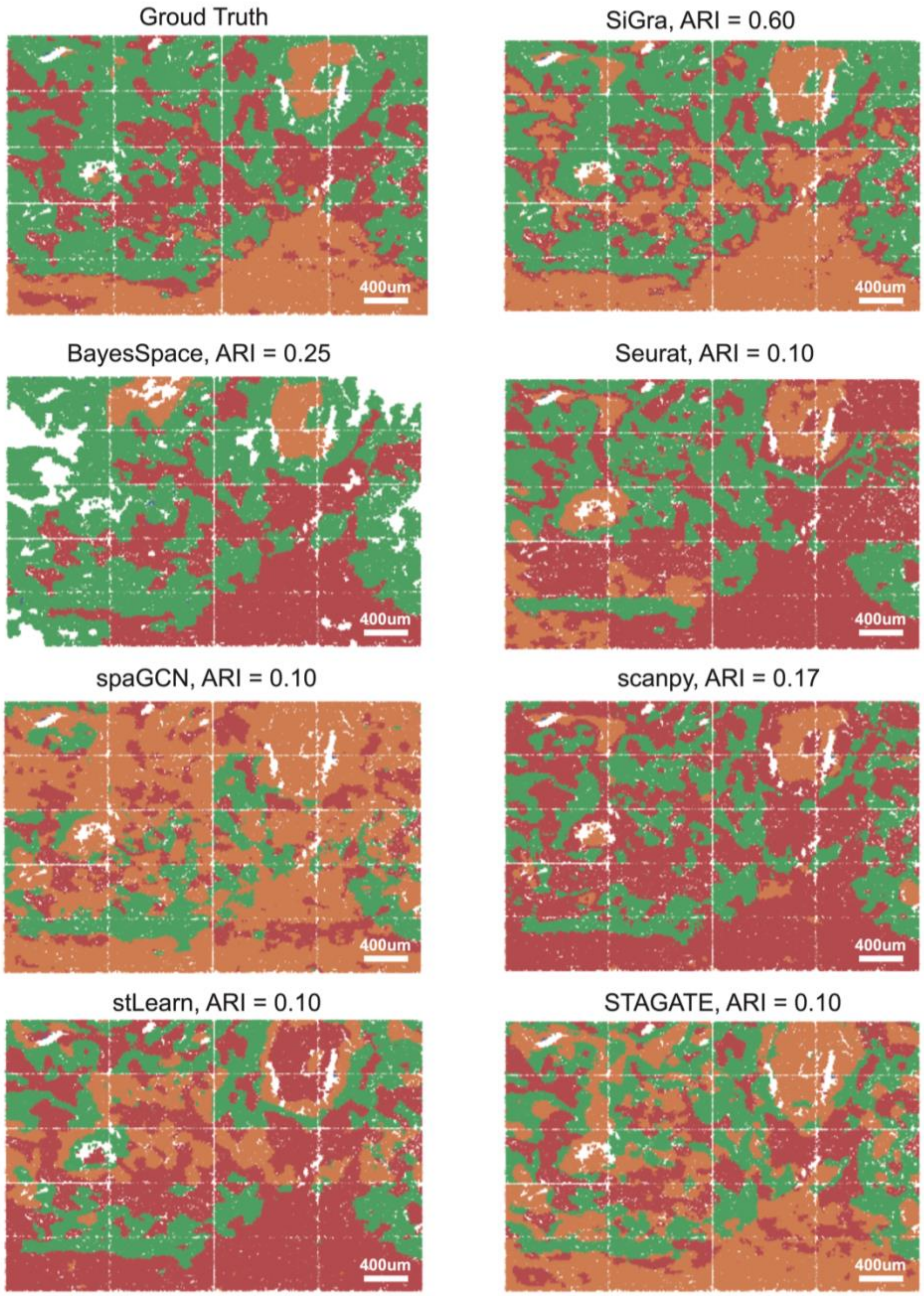

Supplementary Fig. 5: Evaluation and visualization of spatial domains in the NanoString CosMx lung cancer slice.

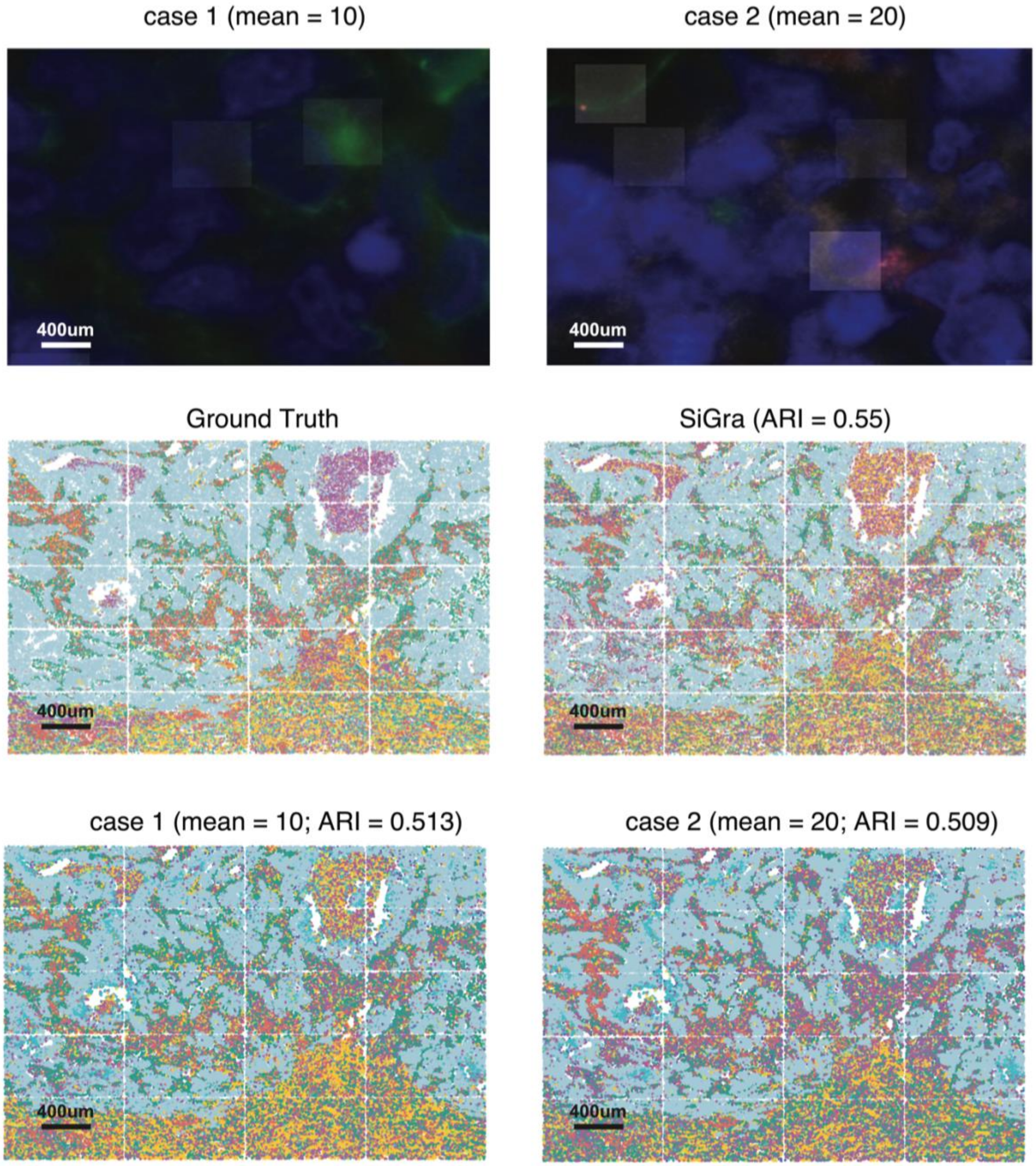

Supplementary Fig. 6: Examination of the effects of autofluorescence on SiGra's performance. Number of cropped images: 12 in each case.

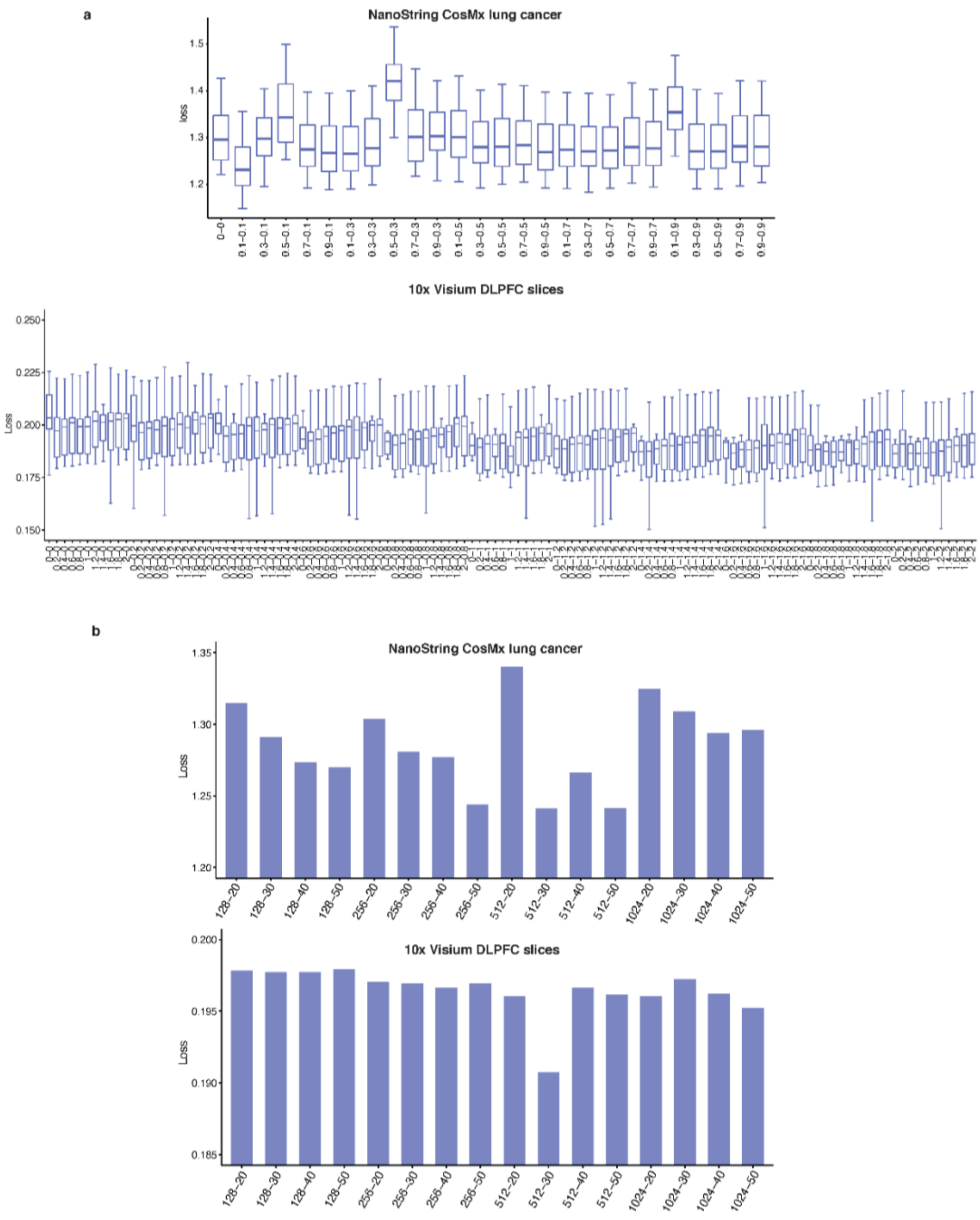

Supplementary Fig. 7: Grid-based hyper-parameter fine turning. a, Identification of the optimal parameters in SiGra's loss function (8 slices from the NanoString CosMx lung cancer dataset and 12 slices from the 10x Visium DLPFC datasets). In the boxplot, the center line, box limits and whiskers denote the median, upper and lower quartiles, and 1.5× interquartile range, respectively. **b,** Identify the optimal latent dimensions of SiGra for NanoString CosMx and 10x Visium data (20 FOVs from the NanoString CosMx lung cancer dataset and 12 slices from the 10x Visium DLPFC datasets). Source data are provided as a Source Data file.

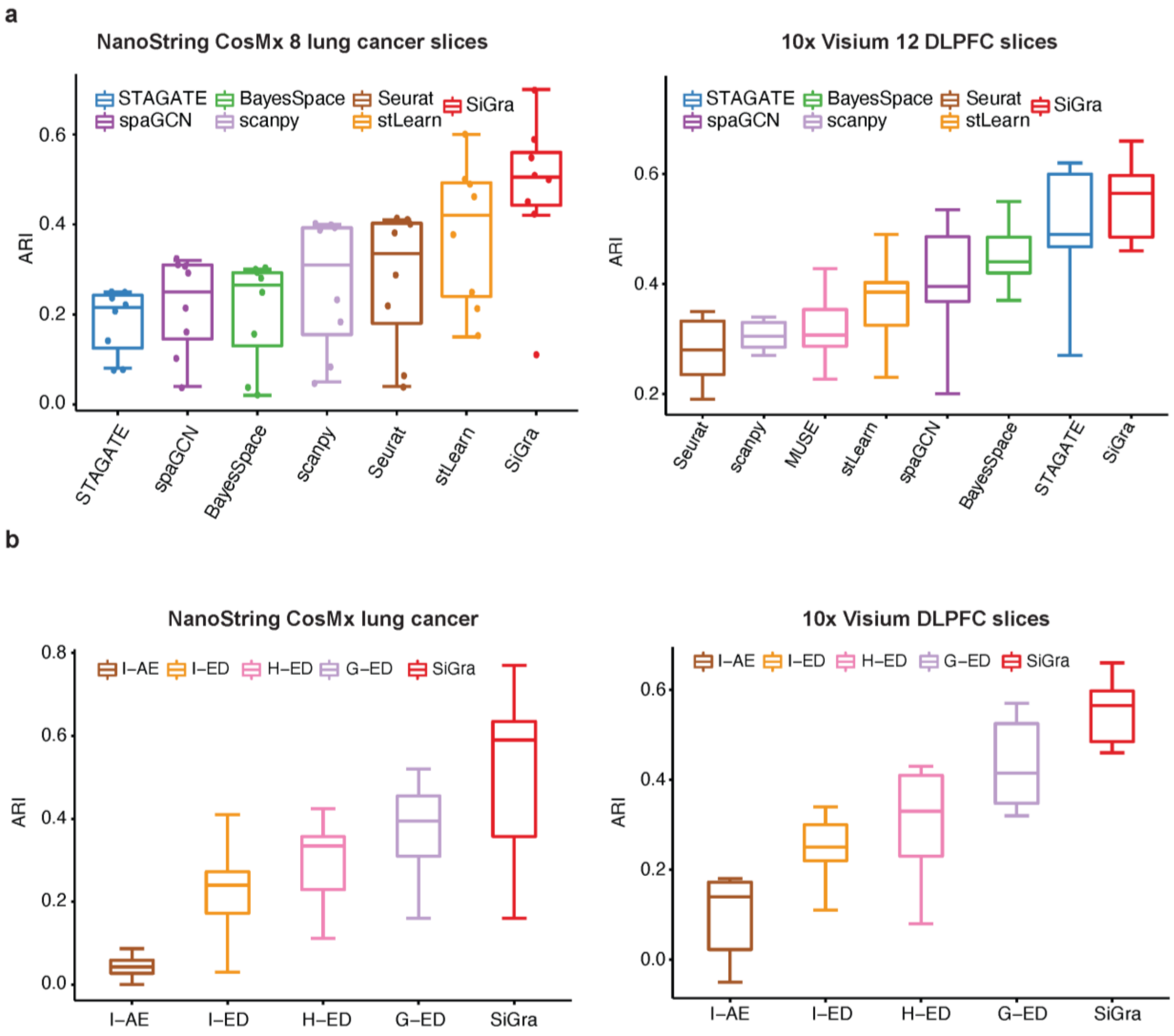

Supplementary Fig. 8: Comprehensive benchmarking and ablation studies. a, Comparisons of SiGra with current available methods on all 8 NanoString CosMx lung cancer slices and all 12 DLPFC slices. In the boxplot, the center line, box limits and whiskers denote the median, upper and lower quartiles, and 1.5× interquartile range, respectively. **b,** Ablation studies for evaluating the contribution of different components in SiGra model. Boxplot shows the ARI scores of ablated models across all the 20 FOVs of lung cancer tissue slice and all 12 DLPFC slices. In the boxplot, the center line, box limits and whiskers denote the median, upper and lower quartiles, and 1.5× interquartile range, respectively. Source data are provided as a Source Data file.

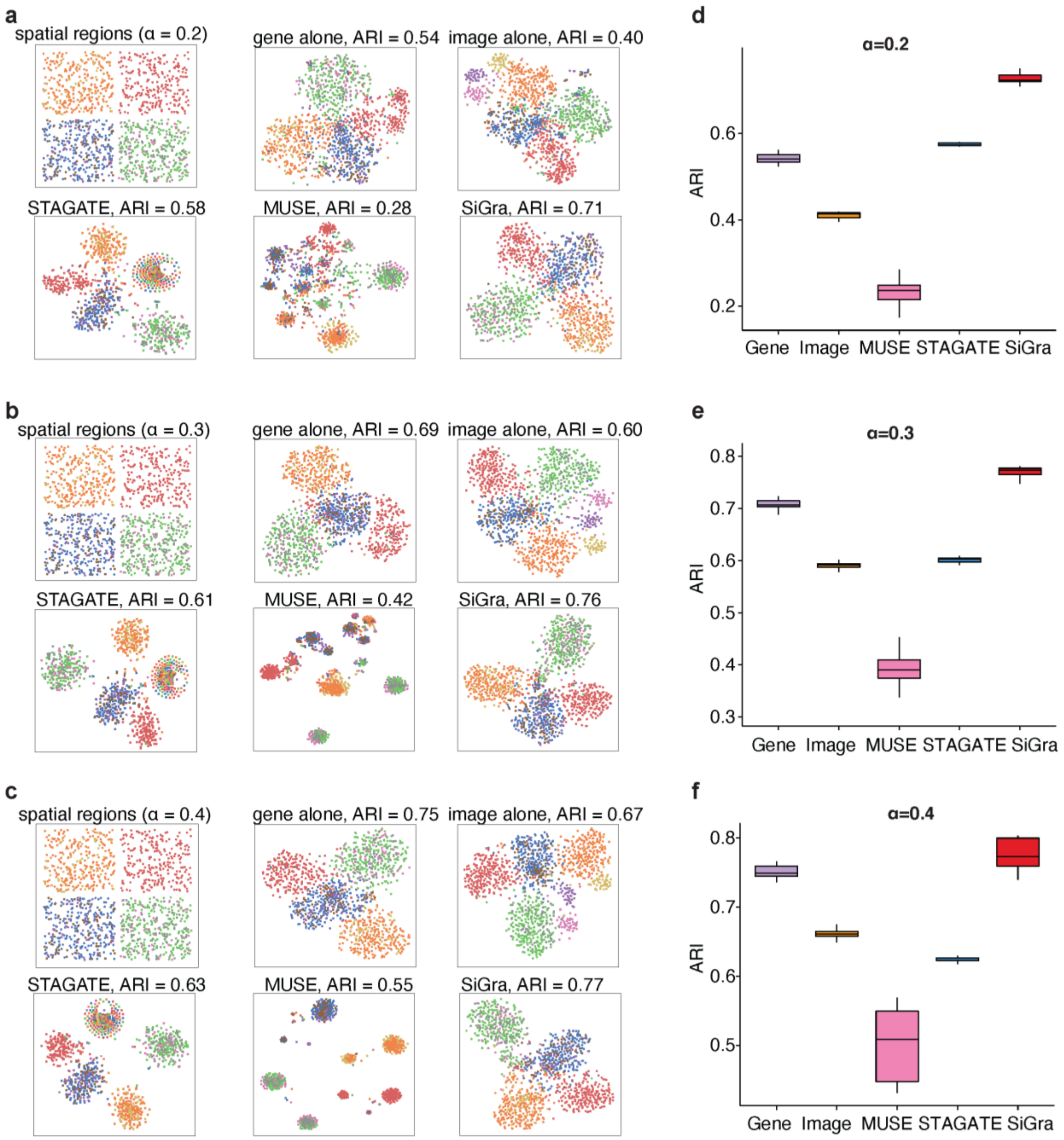

Supplementary Fig. 9: Comparisons with MUSE and STAGATE based on simulation data. (a-c), Visualizations of simulation data with different dropout levels. Spatial domains and domain clusters identified by different methods (STAGATE, MUSE, and SiGra) are shown. Gene-alone and image-alone refer to PCA results based on the single modality data. Different colors refer to the ground truth of cell types in simulation data. **(d-e)**, Accuracy of identifying spatial domains based on 10 simulation replicates over a range of dropout levels (10 samples for each). Clustering accuracy is quantified using ARI. In the boxplot, the center line, box limits and whiskers denote the median, upper and lower quartiles, and 1.5× interquartile range, respectively. Source data are provided as a Source Data file.

SUPPLEMENTARY NOTES

Supplementary Note 1: SiGra improves the quality of single-cell spatial transcriptomics data

Here we demonstrate that the enhanced data by SiGra provides more information than raw data and highlight the necessity of gene expression enhancement for single-cell spatial transcriptomics (SCST) data.

1). NanoString CosMx Lung cancer slice

Specifically, for the lung cancer NanoString CosMx data in Fig. 2, we compared both enhanced data and raw data with existing bulk RNA-seq data. Here we used the bulk RNA-seq data from TCGA lung cancer patients. As shown in **Supplementary Fig. 2a**, the x-axis and y-axis represented the total log-transformed counts per gene in lung cancer slices between the two types of technologies, i.e., SCST and bulk. Each point represented the RNA count for a single gene, averaged across different experimental samples for the corresponding technology. The RNA counts between enhanced SCST and bulk sequencing showed better concordance (cor = 0.631) than that between raw SCST and bulk data (cor = 0.579).

In addition, we also evaluated the potential false discoveries in the L-R associations (**Fig. 3e**) using randomized control. Briefly, we assumed that randomly selected gene pairs from the SCST data were not likely associated and thus used as negative controls. By comparing with these negative controls, the false discovery rate of the selected L-R associations was estimated. Briefly, 10,000 gene pairs were randomly selected, and the corresponding Pearson correlations were calculated as negative control. For each of the L-R pair (**Fig. 3e**), we estimated the false discovery rate accordingly using the FDR values based on the negative controls instead of the Pearson correlations. As shown in the **Fig. 3e**, the y-axis and x-axis referred to the FDR values of each L-R pair in the enhanced and raw data respectively. Across the total 660 L-R interactions, 55 L-R pairs from the enhanced data were statistically significant (FDR < 0.05), whereas 42 L-R pairs from the raw data had FDR < 0.05. There were 28 L-R pairs shared between enhanced data and raw data, indicating enhanced data preserved useful information of raw data. In addition, 27 specific L-R interactions were identified from the enhanced data, while 14 specific L-R interactions were found in the raw data. In **Supplementary Fig. 2b**, we further investigated whether these specific L-R interactions pairs were similar with the shared L-R pairs. For those significant L-R pairs identified from the enhanced data, there were no significant difference between the specific and shared L-R pairs, suggesting that both had similar probability of being true associated L-R pairs. In contrast, the raw-specific L-R pairs were statistically different from the shared L-R pairs, suggesting that the raw-specific pairs were more likely to be false discoveries than the shared L-R pairs. These results indicated that the enhanced data not only enabled to detect more L-R interactions than the raw data, but also the identified L-R pairs were more likely to be true discoveries than those specifically detected in raw data. The data enhancement using SiGra not only improved the sensitivity of L-R interaction detection (identifying more L-R pairs), but also preserved the specificity (the specifically identified L-R pairs that had similar statistical significance as the shared L-R pairs). These results indicated that those raw-specific L-R pairs were more likely to be false discoveries, which could result from noises and the low data quality

in the raw data. Therefore, SiGra improved both the sensitivity (more identified L-R pairs) and the specificity (more true discoveries) for the detection of L-R interactions.

2). MERSCOPE mouse liver data

For the MERSCOPE mouse liver data in **Fig. 4**, we visualized the identified cell clusters and enhanced gene expression of Vwf in **Supplementary Fig. 2c** and **Supplementary Fig. 2d**, accordingly. We also demonstrated the enhanced data quality by comparing with the bulk RNA-seq data from The Tabula Muris Consortium[1].

Supplementary Fig. 2e shows the comparisons between SCST and bulk data for mouse liver MERSCOPE data, where the x-axis and y-axis were the total log-transformed counts. Similarly, each point represents the RNA count for a single gene, averaged across different samples for the corresponding technology. Again, we observed much higher correlation of RNA counts between enhanced SCST and bulk data (cor = 0.854), in contrast with the comparisons between raw SCST and bulk data (cor = 0.800).

In addition, we anticipated that the differential expression analysis also benefited from the enhanced data given its improved data quality. To further verify it, regarding **Fig. 4e**, we used the single-cell RNA-seq data from the Tabula Muris Consortium 2020[2] to identify the differential expression genes (DEGs) in the cell clusters of hepatocytes, periportal hepatocytes, hepatic stellate cells, and endothelial cells. In this way, we then evaluated the overlaps between the scRNA-seq's DEGs and enhanced data's DEGs, as well as the overlaps between the scRNA-seq's DEGs and the raw data's DEGs. As shown in the **Supplementary Fig. 2f**, we identified the overlapped DEGs with scRNA-seq for each cluster. The purple-colored bars represent the number of DEGs shared between scRNA-seq and raw data, and the orange-colored bars represented the number of DEGs shared between scRNA-seq and enhanced data. We also labeled the number of DEGs on the bar plot, for example, for C-1, "12 vs 12" referred to "the shared DEGs between scRNA-seq and raw data" vs "the DEGs of raw data", and "49 vs 59" referred to "the shared DEGs between scRNA-seq and enhanced data" vs "the DEGs of enhanced data". Across different clusters, the enhanced data was shown to recover more dysregulated genes than the original SCST data.

Supplementary Note 2: Interrogation of layer enriched gene markers in enhanced data

Based on the results of DLPFC (**Fig. 5**), we compared the layer-enriched gene markers in our enhanced data with the original study[3] (Maynard et al., 2021). Specifically, we performed the exact statistical analysis in Maynard et al 2021 ("Layer-level gene modeling" and fit 'Enrichment' and 'Pairwise' models) using layer-level enhanced data obtained by SiGra. The variations of gene expressions across layers were examined by two statistical models: **1)** The 'Enrichment' model. Layer-level summarized gene expression result was first fitted using the lmFit and eBayes function from the R package "limma" (version 3.16), after being blocked by the six pairs of spatially adjacent replicates and taking this correlation into account as computed by duplicateCorrelation. Then the Student's t-test statistics was used to compare each layer against the other six using the layer-level data. This resulted in seven sets of Student's t-test statistics (one per layer) with double-sided P values. We focused on genes with positive Student's t-test statistics (expressed higher in one layer against the others) because these are enriched genes rather than depleted genes. **2)** The

‘Pairwise’ model used the same “limma” functions for data processing and taking into account the same correlation structure in addition to using the contrasts.fit function provided by “limma”. Then we also computed the Student’s t-test statistics for each pair of layers. The Student’s t-test statistics with double-sided P values for both ‘Enrichment’ model and ‘Pairwise’ model were provided in Supplementary Table 3. Our results showed that the SiGra enhanced data showed consistent results with the original study[3].

Supplementary Note 3: Reveal spatial domains in single-cell spatial profiles

SiGra can identify spatial domains at various resolutions, depending on the data type and the applications. The spot-level spatial data has a low spatial resolution and consists of mixed cells/cell types in each spot. For example, the spatial resolution of the 10x Visium data is 100µm, measured between the centers of two neighboring spots. For such low-resolution data, SiGra directly and accurately reveals the spatial structures such as the anatomic layers in the DLPFC slices (**Fig. 5**) by clustering the latent-represented spots using Leiden. In contrast, the single-cell spatial data has significantly higher resolution. For example, the spatial resolution of the NanoString CosMx molecular imaging is 52nm, and the summarized gene expression profile based on image segmentation provides single-cell level resolution. SiGra thus can reveal spatial regions at the cellular level (**Fig. 2** and Fig. **3**) and microanatomic level (**Fig. 4**, the identifications of the microanatomic regions in the liver).

Meanwhile, on such high-resolution single-cell spatial data, the regional anatomic spatial structures can be revealed by further summarizing the Leiden clustering results (heterogenous cell types) with a dimensional moving window agglomeration approach. Such approaches have been well-established in spatial data analysis of geographical information systems (GIS) data[4,5] and have recently been used for revealing spatial domains in single-cell spatial data (for example, SSAM[6] by Park et al.). Specifically, the SiGra clustering results were summarized by a circular window of diameter d sliding at both x and y directions across the whole image with a given stride length s. At each stop $C_{i,j}$ with the coordinate $\left(x_i, y_j\right)$, a vector $c_{i,j} \equiv [q_1, \dots, q_t]$ representing the proportions of the SiGra identified clusters (t) covered by the sliding window was calculated. All the stops $\left\{C_{i.j}\right\}$ were recursively merged to k groups $\{a_1, \dots, a_k\}$ by hierarchical clustering according to the cluster proportion vectors $\left\{c_{i.j}\right\}$. These agglomerated groups were defined as the discovered spatial domains. The original slide image was then labeled with the discovered spatial domains according to the coordinate of each stop. In this way, we obtained the spatial domains based on the heterogenous cells identified on the spatial slice. The window radius d used in our work was 100µm, which was consistent with the 10x Visium spatial resolution, with the stride s of 10µm.

The ground truth of the anatomic spatial domains of the NanoString CosMx lung cancer slide was provided by a certificated pathologist at Indiana University Health, Dr. Tieying Hou, according to the IHC images. As shown in **Supplementary Fig. 5**, three spatial domains were identified by Dr. Hou: the tumor region (green), the desmoplasia region (red), and the adjacent normal region (orange). For fair comparisons with other methods, the same spatial moving window agglomeration approach was used. Compared with this ground truth, SiGra achieved an ARI of 0.60, better than other methods including BayesSpace (ARI: 0.25), spaGCN (ARI: 0.10), Seurat (ARI: 0.10), stLearn (ARI: 0.10), and scanpy (ARI: 0.17). These results showed that SiGra obtained reliable spatial domains based on its identified accurate cell identities. It also indicated

that the NanoString CosMx profiled cancer tissue slice was much more challenging given its strong cellular heterogeneity, large cell number, and high-resolution, compared with the 10x Visium profiled normal DLPFC tissues that had well-organized anatomic structure.

To further verify the comparison results, we also tested BayesSpace and spaGCN for direct spatial domain identification of the three domains, without using the moving window agglomeration approach. BayesSpace and spaGCN obtained ARIs of 0.15 and 0.19, respectively. These results further demonstrated that, for detecting large-scale anatomic spatial domains from single-cell spatial data, it was necessary to agglomerate the high-resolution cellular-level clustering results.

Supplementary Note 4: Impact of autofluorescence signals on SiGra

Lipofuscin accumulates in brain tissues during aging or under pathologic conditions, and forms plaques of around 10µm[7]. Such lipofuscin plaques emit autofluorescence signals across major florescent channels used in single cell spatial images. To examine if the extend of lipofuscin and autofluorescence would affect the performance of SiGra, we randomly overlaid simulated autofluorescence signals from plaques of 10µm-by-10µm to all channels in the original image data. The autofluorescence signal intensity was simulated by signals following normal distribution with mean as 10, 20, and 40, respectively. **Supplementary Fig. 6** showed the zoomed-in figures of the images with added lipofuscin autofluorescence signals. The simulated lipofuscin autofluorescence slightly undermined SiGra's performance, from the original ARI (ARI: 0.55) to 0.513 and 0.509 for the added mild (mean signal: 10) or significant (mean signal: 20) autofluorescence signals, respectively. Of note, when the autofluorescence signals was overwhelming (mean signal of 40), the performance of SiGra dropped to 0.41. This simulation experiment suggested that under common experimental conditions, the lipofuscin autofluorescence or other types of autofluorescence would not significantly impact the SiGra performance.

Supplementary Note 5: Hyperparameter tuning of SiGra model

Fine-tuning of λ_1 and λ_2

Regarding the final loss function, we have two hyper-parameters, λ_1 and λ_2, that are used to weight the image-based loss and gene-based loss. The choice of λ_1 and λ_2 are identified based on a grid-search approach. Through the grid-based hyper-parameter fine turning, the optimal parameters are λ_1 = 0.1 and λ_2 = 0.1 for single-cell spatial transcriptomics data; and for 10x Visium data, the optimal parameters are λ_1 = 1 and λ_2 = 1.

The two hyper-parameters, λ_1 and λ_2, are used to balance the contributions of the three encoder-decoders through the image-based loss $L_{M,i}$ and gene-based loss $L_{g,i}$ relative to the hybrid loss $L_{h,i}$. Since the single-cell spatial transcriptomics data and the 10x Visium data are significantly different in terms of the image types (IHC vs H&E images, with different biological meanings of channels), spatial resolutions (single-cell level vs spot level), the coverage of the transcriptome (~1,000 genes vs the whole transcriptome), and the gene expression identification methods (probe-based spatial molecular imaging vs next generation sequencing), we have fine-tuned the two hyper-parameters specifically for each data type.

As shown in **Supplementary Fig. 7a,** we first performed coarse searches to identify the optimal parameter range for each data type, then used a grid-search approach for fine-tuning to determine

the optimal values. The best options for the two parameters were chosen based on the loss evaluation on the validation set (30% of the overall data).

For single-cell transcriptomics data, the coarse search suggested that the optimal solution should be in the range between 0 and 1 for both parameters. Based on the 20 FOVs across the lung cancer tissue, we screened the options for λ_1 and λ_2, ranging from 0.1 to 0.9 with a 0.2 interval. As shown in the figure, the combination of $\lambda_1 = 0.1$ and $\lambda_2 = 0.1$ showed the lowest loss (median: 1.23) across all FOVs. Meanwhile, the combination of $\lambda_1 = 0.5$ and $\lambda_2 = 0.3$ showed the worst loss (median: 1.42).

For the 10x Visium data, the coarse searching suggested that the optimal solution should be in the range between 0 and 2 for both parameters. We chose the best options for the two parameters based on the loss evaluation on the validation set (30% of the overall data). Based on the 12 DLPFC slices, we screened the options for λ_1 and λ_2, ranging from 0 to 2 with a 0.2 interval. As shown in **Supplementary Fig. 7a**, the combination of $\lambda_1 = 1$ and $\lambda_2 = 1$ showed slightly lower loss (median: 0.185) across all slices, while $\lambda_1 = 0$ and $\lambda_2 = 0$ showed the highest loss (median: 0.204) for all slices.

The fine-tuning results further suggested that all three encoder-decoders played important roles in reconstructing the spatial gene expression. For example, for the NanoString CosMx data, although the weights of the losses associated with the image-based encoder-decoder (I-ED) and gene-based encoder-decoder (G-ED) were both 0.1, these two encoders boosted the overall ARI from 0.34 (the hybrid encoder H-ED alone) to 0.57 (SiGra).

Dimension tuning

As shown in **Supplementary Fig. 7b,** we selected the hyper-parameters including the embedding dimensions based on the grid-search approach. Similar to the selection of λ_1 and λ_2, we fine-tuned the dimensions (D_1 and D_2) of the 1st and 2nd layers respectively, based on the loss obtained from the validation set (30% of the overall data). We screened the options for D_1 ranging from 128, 256, 512, 1024, and D_2 ranging from 20, 30, 40, and 50. Specifically, for the NanoString lung cancer slice, the combination of $D_1 = 512$ and $D_2 = 30$ showed the lowest loss (loss: 1.240). For the 10x Visium data, we used the 10x DLPFC slice for screening. The combination of $D_1 = 512$ and $D_2 = 30$ also showed the lowest loss (loss: 0.190).

Supplementary Note 6: Comprehensive benchmarking, ablation studies, and simulation studies

1) Performance benchmarking of SiGra with current available methods.

NanoString CosMx lung cancer slices:

In **Supplementary Fig. 8a,** we included all 8 lung cancer slices and evaluated the ARI scores achieved by different methods, specifically including STAGATE. Across all slices, SiGra obtained higher ARI (median ARI: 0.51) than the other methods, including stLearn (median ARI: 0.42), SpaGCN (median ARI: 0.25), and BayesSpace (median ARI: 0.27). STAGATE presented much lower ARI scores with median ARI only as 0.22. For the slice of lung-6, STAGATE only obtained

ARI around 0.1, whereas SiGra achieved much better ARI (ARI: 0.7). These results showed that SiGra outperformed current available methods in recognizing single-cell spatial data.

<u>*10x Visium data:*</u>

As shown in the **Supplementary Fig. 8a**, we evaluated the ARI scores achieved by different methods, specifically including STAGATE and MUSE. Across all 12 DLPFC slices[3], SiGra obtained higher ARI (median ARI: 0.57) than the other methods, including stLearn (median ARI: 0.39), SpaGCN (median ARI: 0.40), and BayesSpace (median ARI: 0.44). STAGATE presented slightly lower ARI scores with median ARI only as 0.49. On slice 151669, STAGATE obtained lowest ARI (ARI: 0.27), where SiGra achieved much better ARI (ARI: 0.49). Meanwhile, MUSE also presented lower ARI scores with median value as 0.31. The highest ARI that MUSE obtained was 0.43 on slice 151669, and the lowest ARI (ARI: 0.23) was on slice 151576. These results showed that MUSE had modest performance for recognizing the spatially organized brain structures, and SiGra outperformed current available methods in recognizing the organized brain structures.

2) Ablation studies

Here we added ablation studies to investigate the contributions of the transcriptomics-based encoder-decoder (G-ED), the image-based encoder-decoder (I-ED), and the hybrid encoder-decoder (H-ED) on both single-cell spatial transcriptomics (NanoString CosMx SMI) and spot spatial transcriptomics (10x Visium) data. To further demonstrate why SiGra included an image-to-gene encoder-decoder (I-ED) instead of an image-to-image auto-encoder (I-AE), we also compared the performance of the I-AE-based and the I-ED-based ablated models.

We presented the performance of four ablated models, with only one encoder-decoder in each model. As shown in the **Supplementary Fig. 8b: (1) NanoString CosMx SMI.** We first evaluated the adjusted rand index (ARI) scores achieved by the ablated models on the NanoString profiled lung cancer tissue slice (Fig. 2). SiGra obtained the best ARI's across all 20 field-of-views (FOVs) (median ARI: 0.59) than the other ablated models. In contrast, without the other two components, the hybrid encoder-decoder (H-ED) alone only achieved a median ARI of 0.34. The G-ED and I-ED also presented lower ARI scores with median values of 0.40 and 0.24. I-AE obtained a lower ARI (median: 0.04) than I-ED (median: 0.24) across different FOVs. (**2) 10x Visium.** Then we evaluated the ARI scores achieved by the ablated models across the 12 dorsolateral prefrontal cortex (DLPFC) slices. SiGra obtained the highest ARI in all slices (median ARI: 0.57). In contrast, the H-ED presented a lower median ARI score of 0.33. The G-ED and the I-ED achieved median ARIs of 0.41 and 0.25, respectively. I-AE also presented a lower ARI (median: 0.14) than I-ED (median: 0.25). These ablation study suggested that the general imaging features extracted by an image-to-image autoencoder (I-AE) were less relevant to spatial domain detection. The imaging features that were relevant to the spatial gene expression patterns, which were extracted by the image-to-gene encoder-decoder (I-ED), proved to contribute to spatial domain identification. Additionally, the advantage of using an image-to-gene encoder-decoder rather than an image-to-image autoencoder was more significant for single-cell spatial transcriptomics data.

Collectively, these results demonstrate that the superior performance of SiGra is achieved through three encoder-decoder components. The ablated models with only the hybrid encoder-decoder H-ED or the other encoder-decoder alone are not sufficient to achieve comparable performance.

3) Simulation studies

To further verify the performance of SiGra, here we performed the simulation comparisons based on the simulation design of MUSE[8]. The only difference between our simulated data and MUSE's design was that, the simulation data were generated with spatial locations for each domain.

Details of simulation steps were as follows:

1) First, we generated the ground truth of domain regions $l \in \{1, 2, \dots, L\}$, and K different cell types, where L was the number of spatial domains and K was the number of cell types ($K \geq L$). Each domain was a spatial rectangle region $R_l = \{(r_x, r_y);\ s_{x0} < r_x < s_{x1},\ s_{y0} < r_y < s_{y1}\}$. In each domain, we assigned a dominating/major cell type, with several other cell types scattered and mixed with the major one in this spatial domain. For each cell, we randomly generated its spatial coordinate (s_x, s_y), where $s_{x0} < s_x < s_{x1}, s_{y0} < s_y < s_{y1}$. Here we simulated four spatial regions: R_1 = {c-1, c-5, c-6}, R_2 = {c-2}, R_3 = {c-3, c-7, c-8}, R_4 = {c-4, c-9}. The dominating cell types c-1 in R_1, c-2 in R_2, c-3 in R_3, and c-4 in R_4 had more cell numbers than the other cell types {c-5, c-6, c-7, c-8, c-9}. For each dominating cell type, we generated 1,000 cells in their respective spatial region. For the other mixed cell types, we generated 300 cells respectively.

2) Next, the latent representations of gene expression and morphology images features: Z_G, Z_I ($Z_G \in R^m,\ Z_I \in R^m$), were generated following the design of MUSE, where m was the size of the latent dimension. That is, for either Z_G or Z_I, the latent representations Z_i of the i-th cell was simulated using a multivariable normal distribution: $Z_i \sim \sum_l^L \pi_{l,i} MVN(\mu_l, \Sigma_l)$, where L was the total number of the domain regions (L = 4). If cell i belonged to the l-th domain, then $\pi_{l,i}$ =1, otherwise $\pi_{l,i}$ =0. $\mu_l \in R^m$ was sampled from a uniform distribution with $\Sigma_l \in R^{m\times m}$ the identity matrix. Note that the latent gene features Z_G and latent image features Z_I were generated separately, that is:

$$Z_{iG} \sim \sum_l^L \pi_{l,i} MVN(\mu_{lG}, \Sigma_{lG}) \qquad (1)$$

$$Z_{iI} \sim \sum_l^L \pi_{l,i} MVN(\mu_{lI}, \Sigma_{lI}) \qquad (2)$$

3) The raw gene expression and image features were then generated by a linear transformation by $X_G = A_G Z_G + \delta$ and $X_I = A_I Z_I + \delta$, where $A \in R^{p\times m}$ was the random projection matrix from the uniform distribution between [-0.5, 0.5]. δ was the gaussian noise sampled from $N(0, \sigma^2)$. With additional dropouts added as below, the raw gene expression X'_G and image features X'_I were obtained:

$$X'_G = X_G \mathbf{1}[\exp(-\alpha_G X_G) < \eta_G] \quad (3)$$

$$X'_I = X_I \mathbf{1}[\exp(-\alpha_I X_I) < \eta_I] \quad (4)$$

, where $\mathbf{1}[.]$ was the indicator function, which returned 1 if the argument was true, otherwise returned 0. α was the decay coefficient that controlled dropout levels and η was the random value sampled from the uniform distribution between [0,1].

With the simulated data X_G' and X_I', we used them as input for simulation experiments and benchmarking.

Herein, we generated simulation data including both gene-based and image-based features. We chose three settings with different dropout levels for data generation, i.e., α as 0.2, 0.3, and 0.4.

- When α = 0.2, the generated data was visualized as below in **Supplementary Fig. 9a**. As described above, the spatial data consisted of four spatial regions, where each of them was dominated by one major cell type with other scattered types of cells. Meanwhile, the gene-based features and image-based features were shown to contribute to spatial domain identification at certain levels, with ARI as 0.54 and 0.40 respectively. However, MUSE failed to reveal clear spatial domains with ARI only as 0.28. STAGATE revealed the spatial domains with ARI as 0.58. In contrast, SiGra achieved the highest ARI (ARI: 0.71) and revealed much more accurate spatial domains.
- When α = 0.3, the generated data was visualized in **Supplementary Fig. 9b**. Consistently, four spatial domains were generated, with both gene-based features and image-based features contributed to spatial domain identification (ARI: 0.69, 0.60) respectively. Nevertheless, MUSE and STGATE only obtained ARI as 0.42 and 0.61, which were lower than SiGra (ARI: 0.76).
- When α = 0.4, the generated data was visualized in **Supplementary Fig. 9c**. Similarly, both gene-based features and image-based features contributed to spatial domain identification at certain levels (ARI: 0.75, 0.67). SiGra maintained more accurate (ARI: 0.77) than MUSE (ARI: 0.55) and STAGATE (ARI: 0.63) in revealing spatial domains.

In addition, for each setting, i.e., the dropout levels α was 0.2, 0.3, and 0.4, we generated 10 replicated simulation data and obtained the boxplot of ARI scores for gene-only, image-only, MUSE, STAGATE, and SiGra, respectively. As shown in the **Supplementary Fig. 9d**, SiGra reached much higher ARI scores (median: 0.723) than STAGATE (median: 0.573) and MUSE (median: 0.24) when the dropout level α = 0.2. When α was 0.3 and 0.4, SiGra also presented higher ARI (median: 0.77; 0.78) than STAGATE (median: 0.60; 0.62) and MUSE (median: 0.39; 0.51). These simulation results showed that SiGra achieved more accurate identification of spatial regions through leveraging both gene-based information and image-based information.

Supplementary References

1 Single-cell transcriptomics of 20 mouse organs creates a Tabula Muris. *Nature* **562**, 367-372, doi:10.1038/s41586-018-0590-4 (2018).

2 Almanzar, N. *et al.* A single-cell transcriptomic atlas characterizes ageing tissues in the mouse. *Nature* **583**, 590-595, doi:10.1038/s41586-020-2496-1 (2020).

3 Maynard, K. R. *et al.* Transcriptome-scale spatial gene expression in the human dorsolateral prefrontal cortex. *Nat Neurosci* **24**, 425-436, doi:10.1038/s41593-020-00787-0 (2021).

4 Goodchild, M., Haining, R. & Wise, S. Integrating GIS and spatial data analysis: problems and possibilities. *International Journal of Geographical Information Systems* **6**, 407-423, doi:10.1080/02693799208901923 (1992).

5 Shirowzhan, S. & Sepasgozar, S. M. E. Spatial Analysis Using Temporal Point Clouds in Advanced GIS: Methods for Ground Elevation Extraction in Slant Areas and Building Classifications. *ISPRS International Journal of Geo-Information* **8** (2019).

6 Park, J. *et al.* Cell segmentation-free inference of cell types from in situ transcriptomics data. *Nature Communications* **12**, 3545, doi:10.1038/s41467-021-23807-4 (2021).

7 Gray, D. A. & Woulfe, J. Lipofuscin and aging: a matter of toxic waste. *Sci Aging Knowledge Environ* **2005**, re1, doi:10.1126/sageke.2005.5.re1 (2005).

8 Bao, F. *et al.* Integrative spatial analysis of cell morphologies and transcriptional states with MUSE. *Nature Biotechnology* **40**, 1200-1209, doi:10.1038/s41587-022-01251-z (2022).
