# Supplementary material for: SiGra: single-cell spatial elucidation through an image-augmented graph transformer
Source: Nat Commun. 2023 Sep 12;14:5618. doi: 10.1038/s41467-023-41437-w (PMC10497630; doi:10.1038/s41467-023-41437-w)
Supplement: Supplementary file 3 — Reporting Summary [file 41467_2023_41437_MOESM3_ESM.pdf]

nature portfolio

Corresponding author(s): Baijian Yang, Jing Su, Qianqian Song

Last updated by author(s): Jul 21, 2023

Reporting Summary

Nature Portfolio wishes to improve the reproducibility of the work that we publish. This form provides structure for consistency and transparency in reporting. For further information on Nature Portfolio policies, see our Editorial Policies and the Editorial Policy Checklist.

Statistics

For all statistical analyses, confirm that the following items are present in the figure legend, table legend, main text, or Methods section.

n/a	Confirmed	
☐	☒	The exact sample size (*n*) for each experimental group/condition, given as a discrete number and unit of measurement
☒	☐	A statement on whether measurements were taken from distinct samples or whether the same sample was measured repeatedly
☐	☒	The statistical test(s) used AND whether they are one- or two-sided<br>*Only common tests should be described solely by name; describe more complex techniques in the Methods section.*
☒	☐	A description of all covariates tested
☒	☐	A description of any assumptions or corrections, such as tests of normality and adjustment for multiple comparisons
☐	☒	A full description of the statistical parameters including central tendency (e.g. means) or other basic estimates (e.g. regression coefficient) AND variation (e.g. standard deviation) or associated estimates of uncertainty (e.g. confidence intervals)
☐	☒	For null hypothesis testing, the test statistic (e.g. *F*, *t*, *r*) with confidence intervals, effect sizes, degrees of freedom and *P* value noted<br>*Give P values as exact values whenever suitable.*
☒	☐	For Bayesian analysis, information on the choice of priors and Markov chain Monte Carlo settings
☒	☐	For hierarchical and complex designs, identification of the appropriate level for tests and full reporting of outcomes
☐	☒	Estimates of effect sizes (e.g. Cohen's *d*, Pearson's *r*), indicating how they were calculated

Our web collection on statistics for biologists contains articles on many of the points above.

Software and code

Policy information about availability of computer code

Data collection	The single-cell spatial dataset of NanoString CosMx SMI contains 20 FOVs, which is profiled by the CosMx SMI on Formalin-Fixed Paraffin-Embedded (FFPE) samples of the non-small-cell lung cancer (NSCLC) tissue. The dataset (Lung-9-1) and other lung cancer slices are available from https://nanostring.com/products/cosmx-spatial-molecular-imager/nsclc-ffpe-dataset/. We used the Vizgen MERFISH Mouse Liver Map dataset that contains a MERFISH measurement of a 347 gene panel. Sample L1R1 (liver 1, replicate 1) was used and downloaded from https://info.vizgen.com/mouse-liver-data?submissionGuid=da03b470-e111-425a-b6d2-16d34342f4fe , which includes the list of detected transcripts, gene counts per cell matrix, additional spatial cell metadata, cell boundary polygons, and DAPI images. The human dorsolateral prefrontal cortex (DLPFC) 10x Genomics Visium datasets consists of 12 samples. Each of the sample is manually annotated with up to six cortical layers and white matter. Transcriptomics data and hematoxylin and eosin (H&E) images of corresponding tissue sections are downloaded from http://research.libd.org/spatialLIBD/. Source data are provided with this paper.
Data analysis	All the functions mentioned above were implemented in the Python package SiGra, which are available at github (https://github.com/QSong-github/siGra). Other methods including Seurat v4, Scanpy, stLearn, SpaGCN, and BayesSpace are implemented for comparisons. Seurat and Scanpy are implemented based on their provided vignettes. Briefly, for data preprocessing, 3,000 highly variable genes are selected for log normalization, and top 30 principal components (PCs) are calculated for spatial data clustering. BayesSpace is implemented based on their package vignette. Specifically, the input is the top 15 PCs of the log-normalized expression of the top 2,000 HVGs. The nrep parameter is set as 50,000 and the gamma parameter is set as 3. For stLearn, based on its tutorial, the stLearn.SME.SME_normalized() function is performed on raw counts with parameters use_data = "raw" and weights = "physical_distance". The top 30 PCs of the SME normalized matrix are then used for spatial data clustering and visualization. SpaGCN is applied according to its recommended parameters in the package vignette. That is, the top 15 PCs of the log-normalized expression of the top 3,000 spatial variable genes are used for spatial data clustering. 200 epochs are used for identifying and refining spatial domains. The resolution parameter is selected to ensure the number of clustering is equal to the ground truth. To identify the differentially expressed genes (DEGs), the Wilcoxon test from Scanpy package is used. DEGs of each spatial region is

selected with 5% FDR threshold (Benjamin-Hochberg adjustment) and the log2 Folder Change more than 1 (log2 FC > 1).

For manuscripts utilizing custom algorithms or software that are central to the research but not yet described in published literature, software must be made available to editors and reviewers. We strongly encourage code deposition in a community repository (e.g. GitHub). See the Nature Portfolio guidelines for submitting code & software for further information.

Data

Policy information about availability of data

All manuscripts must include a data availability statement. This statement should provide the following information, where applicable:

- Accession codes, unique identifiers, or web links for publicly available datasets
- A description of any restrictions on data availability
- For clinical datasets or third party data, please ensure that the statement adheres to our policy

The single-cell spatial dataset of NanoString CosMx SMI contains 20 FOVs, which is profiled by the CosMx SMI on Formalin-Fixed Paraffin-Embedded (FFPE) samples of the non-small-cell lung cancer (NSCLC) tissue. The dataset (Lung-9-1) and other lung cancer slices are available from https://nanostring.com/products/cosmx-spatial-molecular-imager/nsclc-ffpe-dataset/. We used the Vizgen MERFISH Mouse Liver Map dataset that contains a MERFISH measurement of a 347 gene panel. Sample L1R1 (liver 1, replicate 1) was used and downloaded from https://info.vizgen.com/mouse-liver-data?submissionGuid=da03b470-e111-425a-b6d2-16d34342f4fe , which includes the list of detected transcripts, gene counts per cell matrix, additional spatial cell metadata, cell boundary polygons, and DAPI images. The human dorsolateral prefrontal cortex (DLPFC) 10x Genomics Visium datasets consists of 12 samples. Each of the sample is manually annotated with up to six cortical layers and white matter. Transcriptomics data and hematoxylin and eosin (H&E) images of corresponding tissue sections are downloaded from http://research.libd.org/spatialLIBD/. Source data are provided with this paper.

Human research participants

Policy information about studies involving human research participants and Sex and Gender in Research.

Reporting on sex and gender	The datasets analyzed in this paper do not contain sex information.
Population characteristics	N/A
Recruitment	N/A
Ethics oversight	N/A

Note that full information on the approval of the study protocol must also be provided in the manuscript.

Field-specific reporting

Please select the one below that is the best fit for your research. If you are not sure, read the appropriate sections before making your selection.

[x] Life sciences [ ] Behavioural & social sciences [ ] Ecological, evolutionary & environmental sciences

For a reference copy of the document with all sections, see nature.com/documents/nr-reporting-summary-flat.pdf

Life sciences study design

All studies must disclose on these points even when the disclosure is negative.

Sample size	We included 8 lung cancer single-cell spatial data profiled by Nanostring CosMx SMI. The other 12 DLPFC slices were included in our study. Comprehensive benchmarking on those datasets shows the out-performance of SiGra than the other methods.
Data exclusions	No data were excluded from the analyses.
Replication	Data and findings were reproducible by re-running the analysis.
Randomization	The patients in this study was not based on randomization.
Blinding	Blinding was not relevant to this study.

Reporting for specific materials, systems and methods

We require information from authors about some types of materials, experimental systems and methods used in many studies. Here, indicate whether each material, system or method listed is relevant to your study. If you are not sure if a list item applies to your research, read the appropriate section before selecting a response.

Materials & experimental systems

n/a	Involved in the study
☒	☐ Antibodies
☒	☐ Eukaryotic cell lines
☒	☐ Palaeontology and archaeology
☒	☐ Animals and other organisms
☒	☐ Clinical data
☒	☐ Dual use research of concern

Methods

n/a	Involved in the study
☒	☐ ChIP-seq
☒	☐ Flow cytometry
☒	☐ MRI-based neuroimaging
