# Supplementary material for: SiGra: single-cell spatial elucidation through an image-augmented graph transformer
Source: Nat Commun. 2023 Sep 12;14:5618. doi: 10.1038/s41467-023-41437-w (PMC10497630; doi:10.1038/s41467-023-41437-w)
Supplement: Supplementary file 4 — Description of Additional Supplementary Files [file 41467_2023_41437_MOESM4_ESM.pdf]

Description of Additional Supplementary Files

Supplementary Data 1
Description: DEGs identified for each layer based on raw data and enhanced data of slice 151676.

Supplementary Data 2
Description: DEGs identified for each layer based on raw data and enhanced data of slice 151507.

Supplementary Data 3
Description: Statistical analysis of layer-enriched genes based on enhanced gene expressions.
